# Dangerous drug interactions leading to hemolytic uremic syndrome following lung transplantation

**DOI:** 10.1186/1749-8090-5-70

**Published:** 2010-09-02

**Authors:** Haralabos Parissis, Kate Gould, John Dark

**Affiliations:** 1 Cardiothoracic Department, Royal Victoria Hospital, Grosvernor Rd, Belfast, BT12 6BA, Nothern Ireland; 2 Cardiothoracic Department, Freeman Hospital, High Heaton, Newcastle upon Tyne, NE7 7DN, UK

## Abstract

**Background:**

To report our experience of a rather uncommon drug interaction, resulting in hemolytic uremic syndrome (HUS).

**Methods:**

Two consecutive cases of hemolytic uremic syndrome were diagnosed in our service. In both patients the use of macrolides in patients taking Tacrolimus, resulted in high levels of Tacrolimus.

**Results:**

The first patient was a 48 years old female with Bilateral emphysema. She underwent Single Sequential Lung Transplantation. She developed reperfusion injury requiring prolonged stay. Tacrolimus introduced (Day 51). The patient remained well up till 5 months later; Erythromycin commenced for chest infection. High Tacrolimus levels and a clinical diagnosis of HUS were made. She was treated with plasmapheresis successfully.

The second case was a 57 years old female with Emphysema & A1 Antithrypsin deficiency. She underwent Right Single Lung Transplantation. A2 rejection with mild Obliterative Bronchiolitis diagnosed 1 year later and she switched to Tacrolimus. She was admitted to her local Hospital two and a half years later with right middle lobe consolidation. The patient commenced on amoxicillin and clarithromycin. Worsening renal indices, high Tacrolimus levels, hemolytic anemia & low Platelets were detected. HUS diagnosed & treated with plasmapheresis.

**Conclusions:**

There are 21 cases of HUS following lung transplantation in the literature that may have been induced by high tacrolimus levels. Macrolides in patients taking Cyclosporin or Tacrolimus lead to high levels. Mechanism of action could be glomeruloconstrictor effect with reduced GFR increased production of Endothelin-1 and increased Platelet aggregation.

## Introduction

Extensive clinical use has confirmed that tacrolimus is a key option for immunosuppression after transplantation [1-3]. Tacrolimus as primary immunosuppressant for lung transplant recipient is associated with similar survival and reduction in acute rejection episodes compare with cyclosporine [4].

Haemolytic uraemic syndrome due to cyclosporin or tacrolimus in a lung transplant population is rare. Up to this year there were only few cases of tacrolimus induced haemolytic uraemic syndrome in lung transplant recipients, has been reported.

Out of 680 heart transplants, 65 heart lung transplantations and 378 lung transplantations since the beginning the transplant program, we identified two cases of tacrolimus induced haemolytic uraemic syndrome (0.178%). Both cases were associated with high tacrolimus levels in a background of macrolide administration.

## Case 1

The first reported case was a 48 years old female that had suffered bilateral severe emphysema. She underwent single sequential lung transplantation. Post operatively she developed reperfusion injury requiring prolonged intensive care unit stay.

**Figure 1:**
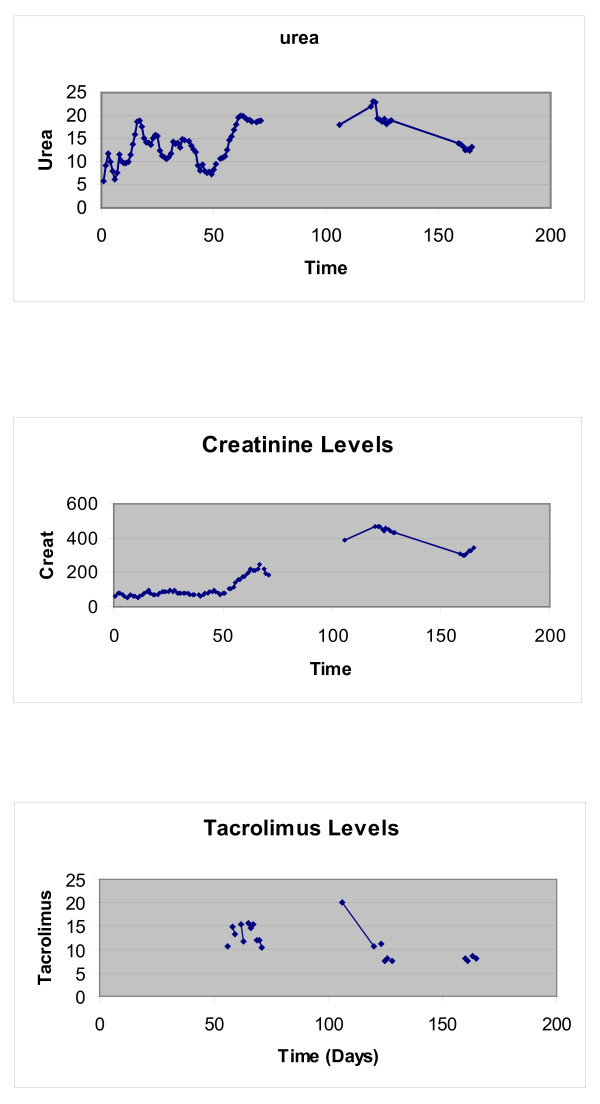
**High Tacrolimus levels corresponding with worsening renal indices (Case 1)**.

She underwent a tracheostomy at the seventh post operative day and an open lung biopsy at the ninth post operative day. The twelfth post operative day she underwent a Laparotomy due to acute abdomen. She was eventually transferred to the ward the 38^th ^post operative day. The baseline urea was 22 mmol/L and Creatinine 250 mmol/L. She was switched (day 52) to tacrolimus 1 mg twice daily due to hirsutism. Following hospital discharge she remained well up to four months where she developed a chest infection and treated with erythromycin. She was admitted to a local hospital (day 120) with worsening clinical picture, uremia (urea 24 mmol/L and Creatinine 490 mmol/L) hemolytic anemia, thrombocytopenia and trough tacrolimus levels of 21 ng/ml (normal 5-15 ng/ml). See Figure 1

Clinical diagnosis of HUS was made. She was treated with plasmapheresis (plasma exchange) daily until the platelet count normalized 8 days later.

## Case 2

The second reported case was a 57 years old, female with a clinical diagnosis of severe emphysema and A1 Antithrypsin deficiency.

She underwent right single lung transplantation. She was discharged home on day 21st. She had a mild renal impairment with the urea of 15 mmol/L and creatinine of 220 mmol/L. She was treated for singles one year later.

She had an A2 rejection 14 months later and a falling FEV1 from 1.26 L to 0.7 L. CT chest (16 months later) showed features consisted with mild Obliterative Bronchiolitis. At this stage she was switched to Tacrolimus 3 mgr BD. By the end of two years and four months following transplantation she has had no further deterioration in lung function and she was on tacrolimus 1 mgr/0.5 mgr, prednisolone 10 mgr and azathioprine 75 mgr daily.

Unfortunately the same period she developed a colonic perforation due to diverticular disease and had a colostomy.

Two years and seven months following her transplantation she was admitted to her local hospital with right side chest pain & breathlessness and right middle lobe consolidation and was treated as pneumonia with amoxicillin and clarithromycin.

The patient was transferred to our service 7 days later with unresolving pneumonia and worsening renal indices (urea from 14 mmol/L to 29 mmol/L and creatinine from 171 mmol/L to 235 mmol/L). The Hemoglobin was 9.6 g/dL, WCC 9.7 x10^3^/mm^3^, Platelets 188.000/mm^3^, urea 28.8 mmol/L, creatinine 239 mmol/L, total bilirubin 19 mmol/L, with normal liver function tests. Arterial Blood Gases on 2 lt of Oxygen showed Ph:7.27, PCO2:4.0 KPa, PO2:10.7 KPa, BE:-12. The tacrolimus level was 7.0 ng/mL. She underwent bronchoscopy & Bronchoalveolar lavage. D-Dimers were not elevated. The patient was allergic to dye therefore a CT pulmonary angiogram was not performed. The clinical picture was considered to be due to an underlying rejection and she was augmented with IV methylprednisolone.

• One day following admission:

Full Treatment for Pulmonary Embolism with tinzaparin. Dialysis was commenced for high Potassium: 6.3 mmol/L and acidosis. High Tacrolimus Levels > 30 ng/mL were also detected. Azothioprine and tacrolimus were stopped.

• Two days following admission:

Falling Haemoglobin to 8.4 g/dL, platelets 126.000/mm^3^

**Figure 2:**
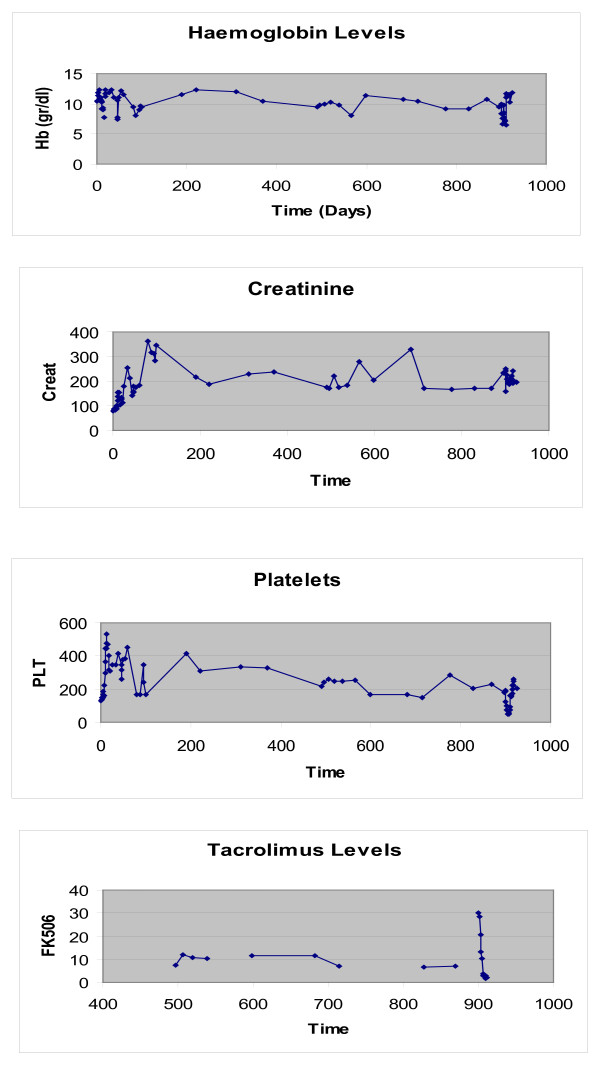
**High Tacrolimus levels corresponding with worsening renal function, anemia and thrombocytopenia (Case 2)**.

• Three days following admission:

Episode of VT. Hb 8.2 g/dL, platelets 99.000/mm^3^, Tacrolimus level 28.6 ng/mL(see Figure 2)

• Four days following admission:

Haemoglobin 7.6 g/dL, platelets 75.000/mm^3^, Serum iron 16 mmol/L (normal 11-29 mmol/L), ferrittin 1571 ng/ml.

• Five days following admission:

Anaemia (Hb: 6.7 g/dL) & Thrombocytopenia (Platelets: 65.000/mm^3^). Blood film showed fragmented red cells. The clotting screen was normal, LDH 1280 U/L, and heparine induced antibodies screen was negative. The diagnosis of HUS was made

• Six days following admission:

Plasmapheresis commenced (Hb: 9.8 g/dL, platelets 52.000/mm^3^)

• Seven days following admission:

Plasma exchange continued, for the next 5 days. At this stage the patient's clinical picture improved and the platelet count has risen to 159.000/mm^3^. In the mean time the immunosuppressant regime had been changed to MMF and rapamycin.

## Discussion

Thrombotic thrombocytopenic purpura- haemolytic uraemic syndrome is an inclusive term describing diverse symptoms of multiple etiologies with common features of thrombocytopenia, microangiopathic haemolytic anaemia, normal clotting screen and also renal, neurological and gastrointestinal involvement [5].

There are two principle mechanisms [5] by which drugs may cause HUS:

A dose related toxicity (eg. cyclosporin) whereby the onset is gradual and an immune mediated reaction (eg. clopidogrel) whereby the onset is explosive and re-exposure produces immediate recurrence. Infection per say is not a significant precipitating factor for post-transplant hemolytic uremic syndrome [5].

A total number of 91 cases of haemolytic uraemic syndrome in adult solid organ transplant recipients were reported by 1996 [6] 90% renal 8% Liver & 2% Heart -Lungs. 96% of the cases occurred within 1 year. In the majority of renal cases and all non-renal transplants HUS was attributed to the use of cyclosporin. Only 4 patients developed HUS while on tacrolimus, including 1 renal transplant recipient and 3 liver transplant recipients. Graft loss due to HUS occurred in 43% of renal transplant recipients. The overall mortality was 13%.

Tacrolimus associated HUS first described by Schmidt et al [7] in a renal recipient in 1991. Until 1999 there were 21 cases reported of tacrolimus associated thrombotic microangiopathy. There were no lung transplant cases in this report.

Various studies have looked at the patient's demographics and also the incidence of HUS amongst transplant recipients [8], [9], [10]: tacrolimus-associated HUS is more frequent in females. The incidence of FK506-associated thrombotic microangiopathy (TMA) is between 1% and 4.7% and the prevalence is between 0.14 and 0.7% [9].

Tacrolimus-associated HUS following lung transplantation was not reported in the literature up until 1999 [11]. Two more cases were subsequently published [12], [13]. Successful resolution of HUS was achieved following conversion from tacrolimus to cyclosporine.

Tacrolimus is a direct glomeruloconstrictor [14]. This effect is dose dependent [15]. It also reduces glomerular filtration rate and increases renal vascular resistance via Endothelin-1. The net effect is ischemia and endothelial cell injury. That triggers inflammation and platelet aggregation, resulting in thrombi and fibrin deposition.

Trough levels of tacrolimus were not predictive for the development of HUS however peak plasma levels of tacrolimus are usually never measured and these could correlate better with tacrolimus -associated HUS [15]. Moreover a reduction in the dose of tacrolimus correlates with an improvement in renal function in most patients [12], [14].

The diagnosis of tacrolimus -associated HUS in the two patients who are the subject of the present report, was supported by an overwhelming clinical picture and clinical follow up. In both patients, high tacrolimus levels were detected following administration of macrolides based antibiotics. They both went on to develop severe microangiopathic haemolysis, thrombocytopenia and renal impairment. They both treated with cessation of tacrolimus and required plasmapheresis, until the platelet count normalized.

All five reported cases in the literature (see Table 1) included patients suffered from emphysema. As to whether there is a relationship between emphysema/a-1 antitrypsin deficiency and the development of HUS in a background of tacrolimus toxicity remains to be seen.

**Table 1:** Literature review of Tacrolimus induced HUS following Lung Transplantation

Case	Age/ Gender	disease	Time to HUS (months)	Dose mgr/day	Level ng/mL	operation	References	year
1	60/F	COAD	9	6/6	16.7	LtLTx	Myers J et al (11)	1999

2	62/M	emphysema	30	6	16.3	RtLTx	Shitrit D et al (12)	2003

3	56/F	emphysema	36	Not reported	Not reported	LtLTx	Boctor FN. et al (13)	2006

4	48/F	emphysema	5	0.5/0.5	20	SSLTx	Own experience	2003

5	57/F	emphysema	19	1/0.5	> 30	RtLTx	Own experience	2003

LtLTx: Left Lung Transplantation

RtLTx: Right Lung Transplantation

SSLTx: Single sequential Lung Transplantation

In conclusion tacrolimus-associated HUS in the lung transplant population is an infrequent entity, and presents with insidious symptomatology requiring high index of suspicion. Furthermore macrolides in patients taking tacrolimus could potentially lead to high levels, with deleterious consequences.

## Competing interests

The authors declare that they have no competing interests

## Authors' contributions

HP conceived of the study, gathered the data and wrote the manuscript, KG made the diagnosis of HUS in those two cases, helped in the design of the study and participated in the sequence alignment, JD participated in the design and coordination and overlooked the progress of the manuscript and advised on valuable amendments. All authors read and approved the final manuscript.

## Consent

Written informed consent was obtained from the patients for publication of those two case reports and accompanying figures. A copy of the written consent is available for review by the Editor-in-Chief of this journal.
